# Dengue in kidney transplanted patients: additions to the puzzle!

**DOI:** 10.1590/2175-8239-JBN-2022-E003

**Published:** 2022-02-02

**Authors:** Vinicius Daher Alvares Delfino, Marilda Mazzali

**Affiliations:** 1 Universidade Estadual de Lodrina, Pontifícia Universidade Católica, Londrina, PR, Brasil.; 2 Universidade Estadual de Campinas, Campinas, SP, Brasil.

Arboviruses are viruses transmitted by arthropods (Arthropod-borne virus) and are so designated not only because of their transmission through hematophagous arthropods, but mainly because of the fact that part of their replication cycle occurs in these insects. The arboviruses that cause disease in humans are members of five viral families: Bunyaviridae, Togaviridae, Flaviviridae, Reoviridae, and Rhabdoviridae^1^.

Dengue virus is an arbovirus of the Flavivirus genus, belonging to the Flaviviridae family. Flaviviruses are single-stranded RNA-encapsulated viruses that use different membrane mediators to penetrate the cell, where in the cytoplasm they use the JAK-Stat complex for viral replication. This group of pathogens is characterized by having immature, mature and not very mature virions in coexistence, facilitating infectivity. In the immunocompetent host, the response to viral infection depends on the release of alpha, beta and gamma interferons, corresponding to the initial phase of the innate immune response. This response is manifested by fever, inflammatory response and infection control within about seven days. In transplant recipients, immunosuppressants, especially calcineurin inhibitors, can limit this interleukin-mediated response, causing a lower frequency of fever. On the other hand, antiproliferative drugs may justify the higher frequency of bone marrow toxicity, especially leukopenia and thrombocytopenia, resulting in a longer hospital stay. Other signs of dengue, such as myalgia, arthralgia and erythema, may have their presentation distorted by the chronic use of steroids^2,3^.

In this case series by Ribeiro et al.^4^ published in this issue of the BJN, attention is drawn to the clinical-laboratorial presentation of longer bone marrow toxicity, with more prolonged thrombocytopenia than in the general population, and a low frequency of fever. The frequency of acute kidney injury (AKI) was 58%, and although most dengue cases were moderate/severe cases (only 4/19, 21% of the patients had dengue without warning signs), there was no mortality or kidney graft loss, with return to baseline renal function in most, but not all patients, during the study period. Nassin et al., in a Pakistani study with the largest number of kidney transplant recipients (Rx) with dengue ever published (102 patients), reported that in 14.3% of those with graft dysfunction, it persisted after six weeks of follow-up^5^.

AKI, which is frequent in cases of dengue in renal transplant patients, should be carefully evaluated, since the reduction in glomerular filtration may be a consequence of systemic hemodynamic imbalance, tissue damage by the virus, or acute rejection episodes, due to the immunomodulation induced by the viral infection or reduced immunosuppression in more severe viral conditions. In this series, there was no case of acute graft rejection. In another series of dengue in kidney transplant patients, the reported incidence of acute rejection was very low, and a recent systematic review on the subject pointed out that graft loss occurs infrequently, and it is usually not associated with renal rejection, but to other concurrent factors^6^.

The paper by Ribeiro et al. adds considerable knowledge about the behavior and management of dengue infection in kidney transplanted patients who require hospitalization in our environment. The clinical and laboratorial workup, although with some peculiarities, is quite similar to that of the general population (Weerakkody et al. reported that kidney transplanted patients who had dengue in the early postoperative period are at risk of more severe disease and unfavorable outcomes^6^); in all RTx, the presence of fever and/or viral-like condition associated with cytopenia (leuko- or thrombocytopenia) should motivate the inclusion of dengue in the differential diagnosis; due to cytopenia, it may be necessary to reduce immunosuppression in a good number of cases; the treatment usually given to those affected in the general population (symptomatic drugs, volume expansion, removal of diuretics - if possible and if necessary - and avoiding nephrotoxic agents) seems to prevent mortality and enable the recovery of renal function in transplant patients affected by dengue similar to those in this study.

A word of caution regarding the return of renal function to baseline in most transplant patients studied is the need for an individualized approach. RTx should be considered as patients with Chronic Kidney Disease (CKD) at different stages and who are at high risk of progression to dialysis and higher mortality^7^ (in the present series, although the patients’ eGFR was not reported, the mean baseline creatinine was 1.74 mg/dL ± 0.61, which points to CKD in stages 3 or more in a significant portion of these patients).

In a series with more than 100,000 patients who had in-hospital AKI, (~17,000 with and 88,000 without ARF), whom Heung et al., followed for one year after the acute episode, demonstrated that the risk of progression to stage 3 CKD increases with the severity of the episode, but the risk is already significantly increased even in patients who had mild elevations in serum creatinine levels which, returned to normal within two days: RR of 1.43 (95% CI, 1.39-1.488).

The fact that a number of transplant recipients in this study already had elevated baseline serum creatinine may characterize some AKI episodes as acute-on-chronic kidney disease, where the trend towards progression to renal chronicity is even higher^9^, which is why for these - and for all patients, transplanted or not, who presented an episode of AKI - long-term follow-up is carried out so that, if necessary, there is prompt establishment of kidney and cardioprotective measures.

The possibility of dengue transmission through kidney transplantation is well documented, but there is no specific guidance for screening for this virus in kidney donors, leaving the suggestion that, in kidney donors from very high prevalence regions, screening for the virus should be considered^10^.

Since Brazil is an endemic country for dengue infection and because the national series of dengue fever in patients undergoing transplants are relatively small, national multicenter studies on this topic would be highly desirable.
